# Decellularized allografts as an alternative for reconstruction of large inferior alveolar nerve defects: a systematic review

**DOI:** 10.4317/medoral.25647

**Published:** 2022-10-16

**Authors:** Gustavo Matus, Juan Pablo Aravena, Dylan Mariño, Sven Eric Niklander

**Affiliations:** 1 Cathedra of Maxillofacial Surgery and Traumatology, Faculty of Dentistry of Universidad Andres Bello, Viña del Mar, Chile; 2 ORCID: 0000-0003-0966-7576. Departament of Morphology, School of Medicine, Universidad Andres Bello, Viña del Mar, Chile; 3 ORCID: 0000-0002-1089-8446. Departament of Morphology, School of Medicine, Universidad Andres Bello, Viña del Mar, Chile; 4 School of Dentistry, Universidad Andres Bello, Viña del Mar, Chile; 5 Unit of Oral Pathology and Medicine, Faculty of Dentistry of Universidad Andres Bello, Viña del Mar, Chile

## Abstract

**Background:**

Inferior alveolar nerve (IAN) injuries are a clinical problem with devastating consequences, causing temporary or permanent paresthesia, significantly affecting the patient's quality of life. Despite morbidity, side effects and controversy regarding its results, autologous nerve grafting is still the main treatment for these type of lesions. However, due to advances in knowledge about nerve damage and with the aim of preventing the described problems of autografts, new treatment alternatives based on decellularized allografts have emerged. The aim of this systematic review was to evaluate the reported efficacy of decellularized allografts for the treatment of IAN damage.

**Material and Methods:**

We performed a systematic search in Pubmed, Scopus and Web of Science databases following the PRISMA guidelines. Cohort studies, randomized or non-randomized clinical studies, prospective or retrospective studies, without age limits and language restriction that included human subjects who received decellularized allograft as treatment for IAN damage were included.

**Results:**

Six articles met the inclusion criteria and were included for data analysis. In all 6 articles, resolution of IAN damage was observed in more than 85% of patients after a 12-month follow-up period, and in 2 of them, complete resolution was observed in 100% of their patients at longer follow-ups.

**Conclusions:**

Decellularized allograft appears to be a promising alternative to resolve IAN lesions, without requiring a nerve autograft procedure. However, more randomized clinical trials are needed to validate adequate treatment modalities with decellularized allografts.

** Key words:**Decellularized allograft, alveolar inferior nerve, allograft, injury.

## Introduction

The inferior alveolar nerve (IAN) is one of the most important and voluminous terminal sensory nerves of the mandibular nerve (V3) of the trigeminal nerve. It runs inferiorly and anteriorly and penetrates the mandibular canal. The latter can present two dispositions. In the most frequent, the IAN runs to the mentonian foramen, dividing into two terminal branches, the mentonian nerve and the inferior dental plexus. The second arrangement is more infrequent, where the nerve divides into two terminal branches at the entrance of the mandibular canal; the mentonian nerve and the inferior dental nerve. Both arrangements innervate the buccal and vestibular mucosa, the vermilion of the lower lip and the cutaneous skin overlying the chin region (1,2).

Injuries to the IAN are a clinical problem with devastating consequences, as they affect the function of the nerve involved and may cause temporary or permanent paresthesia. This significantly affects the patient's quality of life, altering speech, taste, chewing, tooth brushing and the ability to maintain lip competence for food retention and swallowing (1,3-7), resulting in severe disability with important social, personal and psychological effects (4-6,8-13).

According to Seddon's classification there are three types of nerve injury: (i) neuropraxia, where nerve conduction is interrupted at the site of injury preserving the anatomy of the nerve and axon, (ii) axonotmesis, which corresponds to complete interruption of the axon and myelin sheath preserving the endoneurium, (iii) neurotmesis, where complete interruption of the axon and its myelin sheath is evident, without preservation of the endoneurium, preventing spontaneous regeneration (14-17)

IAN lesions can be caused by a great variety of injuries, being the extraction of impacted teeth the most frequent cause, corresponding to 22% of the cases (18). Of these, 25% do not achieve complete recovery during the first year, and 0.9% remain with permanent alterations (19). Other causes that can injure this nerve include the placement of dental implants, orthognathic surgical procedures (6), removal of benign or malignant tumors (6,20), endodontic therapy, injections of local anesthesia and as a direct consequence of maxillofacial trauma and/or surgical interventions for its repair (3).

There are several treatment options for peripheral nerve injuries when there is a loss of nerve continuity at both ends. When a tension-free repair is desired, direct neurorrhaphy should be the procedure of choice (1). However, if it is not possible to perform this technique, the first option is the use of an autologous nerve graft (1,5,6,8), considered the gold standard for nerve grafts. This graft acts as a scaffold that does not produce immunologic reactions and provides neurotrophic factors and Schwann cells, both important for axonal regeneration. For this procedure, donor tissue is usually obtained from the sural nerve and/or the greater auricular nerve (1,5,6,8,14,15,17,18,21), reporting a range of nerve recovery between 87.3% and 100% (22,23). However, this technique is associated with high donor site morbidity, as it requires a secondary surgical procedure to remove the donated nerve tissue (1,5,6,8,15,24,25). Because of the need to minimize autograft complications, such as the risk of neuromas formation, cutaneous scarring, and loss of sensation, the use of conduits as scaffolds to bridge nerve gaps without the interference of a nerve graft has been explored (8,15,17,26). However, this technique has limited applications due to variability in the reported results and its difficulty to be used in small nerve gaps (1,5,8,14,15,19). Other alternative for the treatment of peripheral nerve defects are nerve allografts (4,15,17). These can cover a nerve gap of up to 70 mm in length, and due to the neurotrophic effect they provide, they seem to be more effective than conduits. In addition, they do not need a donor site, thus have a reduced morbidity compared autologous nerve grafts (4,15,20).

In recent years, non-immunogenic nerve allografts have been used with promising results (8,15,20). The term non-immunogenic refers to decellularized nerve allografts, which retain the nerve tissue framework but are inert to the body, since they were previously processed (4,8,17,20). The process of forming these allografts consists of repeated freeze-thaw cycles, exposure to radiation, prolonged storage in University of Wisconsin cold solution, and decellularization with detergents (8,17). The resulting processed allograft retains the native architecture within the original nerve fascicle and epineural scaffold, which comprises extracellular matrix proteins (laminin, fibronectin, and glycosaminoglycans) (8,20). These proteins, in addition to the native microscopic structure, provide natural axonal growth signals for guided growth, which are not currently found in hollow tube conduits (20).

Because the studies reported to date are few and heterogeneous, this study aims to analyze and synthesize the information reported in the scientific literature through a qualitative systematization, in relation to the use of decellularized allografts in IAN defects, and their application as a promising alternative for optimal sensory recovery in the maxillofacial area.

## Material and Methods

- Study design

The following review was performed following the recommendations from PRISMA. The research question to be answered was: "Are decellularized allografts an effective alternative for the reconstruction of nerve defects associated with the inferior alveolar nerve?".

- Eligibility Criteria

The eligibility criteria used in the selection of studies were: full text available, no language restriction, studies reporting the use of decellularized allografts for inferior alveolar nerve reconstruction, cohort studies, clinical studies (randomized or nonrandomized), prospective, comparative and retrospective studies without age limits.

Animal studies and articles reporting the use of decellularized allografts in other nerves unrelated to the research question, narrative reviews, systematic reviews, and *in vitro* or animal studies were excluded.

- Sources of information

To identify potentially relevant articles, MEDLINE/Pubmed, Web of Science and Scopus bibliographic databases were used. Authors G.M.M and J.P.A conducted the search independently between May 1 and June 4, 2021.

- Search strategy

An electronic search was performed based on the research question. The search term used was ("Allografts"[Mesh]) AND "Mandibular Nerve"[Mesh]) OR "Mandibular Nerve Injuries"[Mesh]. In addition, a manual search was performed.

- Article selection

The selection of articles was conducted independently by two reviewers (G.M.M and J.P.A). The main data was exported to Mendeley reference manager. Two reviewers independently analyzed titles and abstracts and identified articles eligible for full review. Disagreements were resolved under consensus and discussion by the two reviewers with a third reviewer who acted as a judge to settle any disagreements (D.M.R).

- Data extraction

Several variables were considered, which were tabulated in an Excel spreadsheet and presented as Tables and/or Figures.

- Risk of bias

To assess the risk of bias of the included studies, we used the Newcastle-Ottawa scale, which allowed us to analyze and calculate a quality score for each selected manuscript based on three main components: (a) selection of the study groups, (b) comparability of the groups, and (c) assessment of the outcome or exposure. The minimum score corresponds to 0 and the maximum to 9, the latter representing the highest methodological quality.

## Results

**Figure 1:**
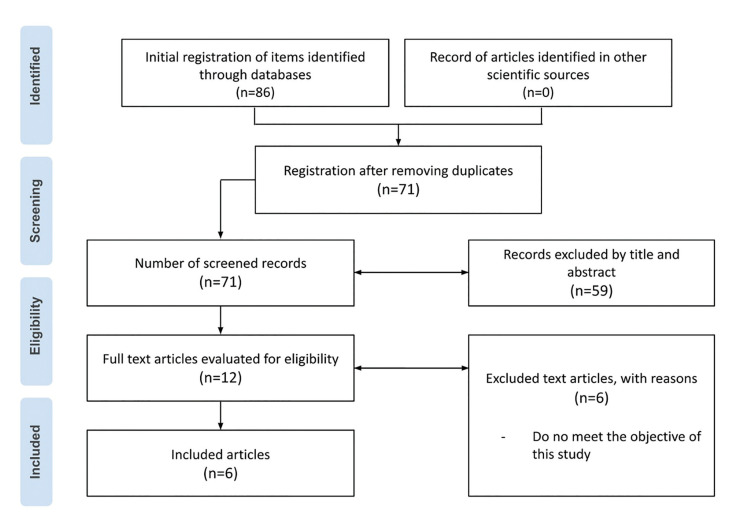
PRISMA flow diagram.

Initially, we identified 86 potential articles, 15 of which corresponded to duplicates and were eliminated. The remaining 71 studies were subjected to title and abstract review, which left a total of 12 potential manuscripts for full-text evaluation. Finally, 6 studies did not meet the inclusion criteria, so 6 articles were included for analysis (Fig. 1).

The studies included in our study were published between the years 2011 - 2020. Six came from the United States. In terms of study design, 1 study corresponded to a case report, 3 to retrospective cohort studies, 1 to a case-control study, and 1 to a case series study. In 4 of the 6 studies was a predominance of females over males (4,5,27,28) and the age range was between 9 and 67 years (6) (Table 1).

The most common cause of IAN injury was ameloblastoma resection (in 3 out of the 6 articles) (4,20,27), followed by complications related to molar exodontias (5,28), post implant installation iatrogenesis and resection of other tumors (6) (Table 2).

**Table 1:**
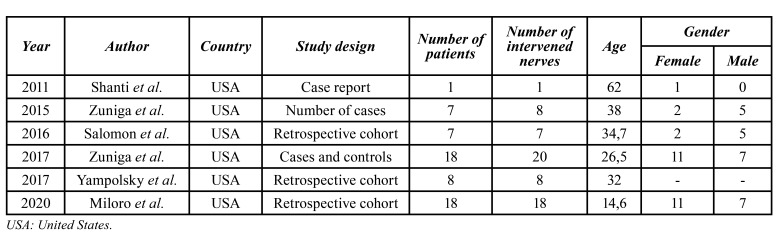
Demographics of the included studies

**Table 2:**
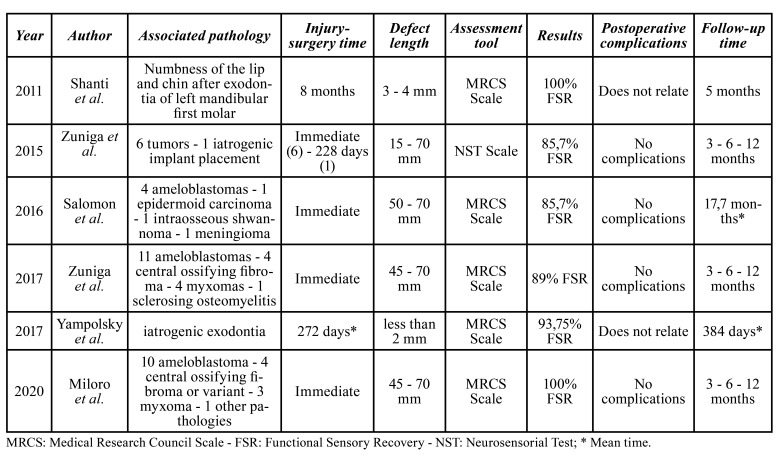
Main summary of the included studies.

All included studies reported the use of decellularized allograft from AxoGen Inc (Alachua FL). Regarding the time interval between the lesion and the surgical procedure, in 3/6 articles the IAN reconstruction was immediate (4,20,27), in 2/6 after 8 and 9 months (5,28), and in one study they reported two time periods, immediate reconstruction and after a period of 8 and 9 months (6). Regarding the size of the nerve defect, the gaps ranged from 2 mm (6) to 70 mm (4,6,20,27).

To determine functional sensory recovery (FSR), in 5/6 articles the Medical Research Council System (MRCS) scale was used as the preferred classification system (4,5,20,27,28), while in 1/6 the Neurosensory Test (NST) (6). Regarding postoperative results, in 2/6 articles FSR was achieved by 100% of their patients (*n*=19) (4,28). Zuniga *et al*. achieved 85.7% recovery, with only 1 patient not achieving FSR (*n*=7) (6), as did Yampolsky *et al*. and Salomon *et al*. with 93.7% (*n*=8) and 85.7% (*n*=7) success respectively (5,20). In the remaining study, only 11% of patients achieved RSF (*n*=18) (27). Follow-up time ranged from 3 months (4,6,27) to 17.7 months (20). No postoperative complications associated with the nerve graft were reported (Table 2).

- Risk of bias

**Figure 2:**
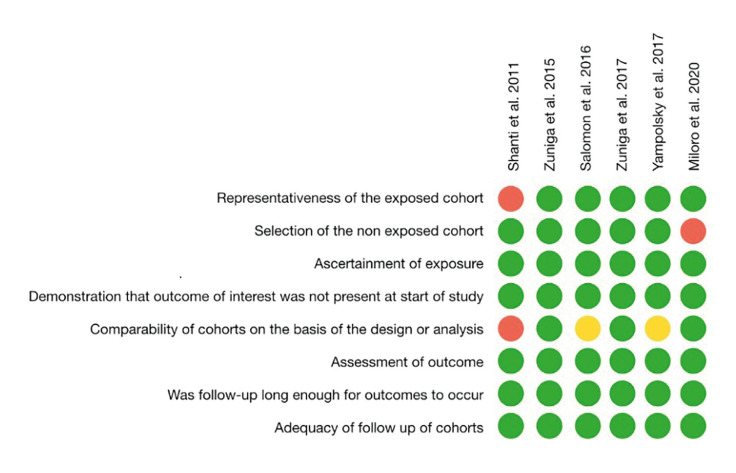
Risk of bias evaluation of each study.

All studies, except of one (4), determined how the selection of the individuals was performed, so the selection item presented low risk of bias. The results were adequately reported in all studies. One article lacked comparability based on design and analysis (28). The overall risk of bias scheme is presented in Fig. 2.

## Discussion

The processed nerve allograft (PNA) is a promising option for IAN reconstruction, since unlike the autograft (considered the gold standard), has greater advantages in terms of morbidity, biocompatibility, convenience of use and supply abundance (29). Currently, Avance Nerve Graft (Axogen Inc, Alachua, FL) is the only PNA available in the market (30). This PNA consists of an extracellular matrix scaffold created from donated human peripheral nerve tissue that has been predegenerated, decellularized and sterilized (1). Decellularization and sterilization of the allograft significantly reduces the risk of immune rejection and thus eliminates the need for immunosuppressive therapy (1,20). The allograft preserves the nerve architecture and extracellular matrix microenvironment, which in turn favors natural guided nerve regrowth, whereas conduits do not have these characteristics (1,20).

This review suggests a positive result in surgical therapy with PNA as treatment alternative for IAN damage, even in patients who underwent chemo and radiotherapy prior to surgery after the excision of a malignant lesion (4). In all studies, a significant improvement was observed in more than 85% of the patients (4-6,20,27,28) and in 2/6 studies a resolution was achieved in 100% of the patients (4,28). Regarding the improvement of the lesions, only 1/6 study reported 1 patient without improvement, nevertheless, this patient evidenced a therapeutic failure in the bone graft that was performed (20). This suggests that the treatment failure was due to infection of the bone graft and not due to the nerve allograft used.

The mean post-surgical follow-up time ranged from 3 to 17.7 months. Zuniga *et al*., and Miloro *et al*., observed a greater recovery rate after 12 months compared to the results obtained after 3 or 6 months (4,6,27). Salomón *et al*. had the longest follow-up time (17.7 months), observing an improvement in almost all their patients during this period, except for the therapeutic failure of the patient with superinfection of the bone graft (20). This suggests that time is important in the resolution of IAN lesions treated with PNA.

Ziccardi *et al*. considered the time elapsed since the initial injury as an important factor for success. If the time elapsed between injury and surgery is excessive, exceeding a time limit of up to 10 weeks, it could be a contraindication for surgical intervention with ANP, since repairs that are completed before this period of time have a better prognosis (31). Nevertheless, this is not clear. Shanti *et al*., intervened patients 8 months after the initial surgery causative of the nerve defect, obtaining S3 results on the MRCS scale (please see Table 3 for more details on the MRCS scale) (28). Yampolsky *et al*, reported a time range between initial injury to surgery of 2 to 923 days, obtaining successful results with 93.75% of the patients, without detailing which time interval was less successful (5). On the other hand, Zuniga *et al*. reported that one of their patients had a lesion-treatment interval of 228 days which was considered the cause for, obtaining a "severe" result in the neurosensorial test (please see Table 4 for more details on the neurosensorial test) (6), Thus, it is not possible to determine if time elapsed is a significant determinant for treatment success.

**Table 3:**
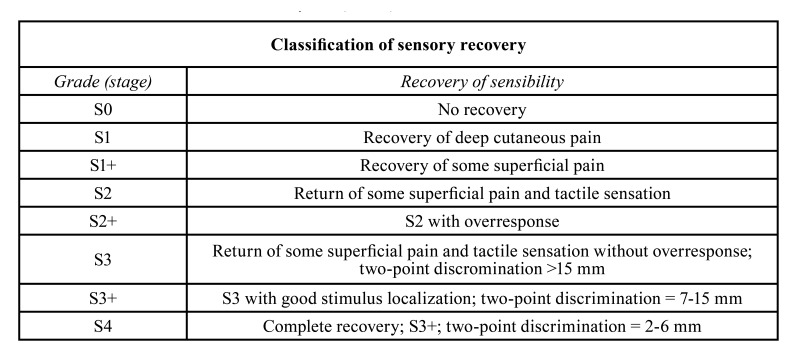
Medical Research Council Scale System (MRCS).

**Table 4:**
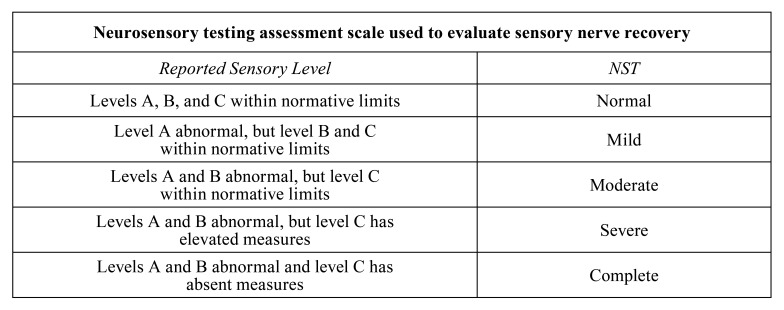
Neurosensory Test (NST) Level A test, spatiotemporal sensory perception with brush- stroke, directional sensitivity, and static 2-point discrimina- tion; level B test, contact detection with Semmes-Weinstein monofilaments; level C test, pain threshold and tolerance using an algometer, thermode, or sharp instrument.

Regarding the length of the defect, Beris *et al*. considered it as a significant factor affecting the allograft result, highlighting that smaller gaps have better results (15). Zuniga *et al*. and Miloro *et al*. described positive results in 89% and 100% of the patients with gaps of 45 mm and 70 mm, respectively (4,6). Salomón *et al*. reported the treatment of six 70 mm and one 50 mm gaps, with a success rate of 85.71% (20). Yampolsky *et al*. reported cases with gaps of less than 2 mm, obtaining positive results in 93.75% of patients (5) and, Shanti *et al*. treated gaps of 3 to 4 mm obtaining S3 results on the MRCS scale (Table 3) (28). According to these results, in gaps smaller than 70 mm, the success of PNA seems not to be significantly affected by the defect length. None of the studies assess PNA treatment for defects larger than 70 mm (Table 2).

Given the positive results of decellularized allograft, we can compare its advantages and disadvantages versus autologous nerve graft, which is considered the gold standard (5,27,28). The latter is reported to have a success rate ranging from 87.3% to 100% (6), similar to the one reported when using decellularized allograft (85.7% to 100%). However, the latter has the advantage of not generating donor site morbidity, as does the autograft, which is mainly extracted from the greater auricular nerve or sural nerve (4-6,27). This can generate sensory loss, neuroma formation or neuropathic pain (28).

The small number of primary randomized studies describing the use of decellularized allografts, the limited availability of clinical information, and the fact that most of the available studies are from the same authors, limit the conclusions that can be drawn from these results. In addition, the publications included are mostly case report or case series.

## Conclusions

Despite the scarce report of primary studies, processed nerve autografting appears to be an effective and promising alternative to achieve positive results of inferior alveolar nerve injuries involving small or wide gaps, without requiring an additional procedure to remove a healthy nerve located elsewhere in the body.

## References

[1] Ducic I, Yoon J (2019). Reconstructive Options for Inferior Alveolar and Lingual Nerve Injuries After Dental and Oral Surgery: An Evidence-Based Review. Ann Plast Surg.

[2] Buch HA (2011). Clinical anatomy of inferior alveolar nerve block anesthesia. Clin Anat.

[3] Bagheri SC, Meyer RA, Khan HA, Steed MB (2009). Microsurgical repair of peripheral trigeminal nerve injuries from maxillofacial trauma. J Oral Maxillofac Surg.

[4] Miloro M, Zuniga JR (2020). Does Immediate Inferior Alveolar Nerve Allograft Reconstruction Result in Functional Sensory Recovery in Pediatric Patients?. J Oral Maxillofac Surg.

[5] Yampolsky A, Ziccardi V, Chuang SK (2017). Efficacy of Acellular Nerve Allografts in Trigeminal Nerve Reconstruction. J Oral Maxillofac Surg.

[6] Zuniga JR (2015). Sensory Outcomes After Reconstruction of Lingual and Inferior Alveolar Nerve Discontinuities Using Processed Nerve Allograft-A Case Series. J Oral Maxillofac Surg.

[7] Tay ABG, Zuniga JR (2007). Clinical characteristics of trigeminal nerve injury referrals to a university centre. Int J Oral Maxillofac Surg.

[8] Panagopoulos GN, Megaloikonomos PD, Mavrogenis AF (2017). The Present and Future for Peripheral Nerve Regeneration. Orthopedics.

[9] Wojtkiewicz DM, Saunders J, Domeshek L, Novak CB, Kaskutas V, Mackinnon SE (2015). Social impact of peripheral nerve injuries. Hand.

[10] Becker SJE, Makanji HS, Ring D (2012). Expected and actual improvement of symptoms with carpal tunnel release. J Hand Surg Am.

[11] Guse DM, Moran SL (2013). Outcomes of the surgical treatment of peripheral neuromas of the hand and forearm: a 25-year comparative outcome study. Ann Plast Surg.

[12] Novak CB, Anastakis DJ, Beaton DE, Mackinnon SE, Katz J (2010). Relationships among pain disability, pain intensity, illness intrusiveness, and upper extremity disability in patients with traumatic peripheral nerve injury. J Hand Surg Am.

[13] Ring D (2013). Symptoms and disability after major peripheral nerve injury. Hand Clin.

[14] Jones RHB (2010). Repair of the trigeminal nerve: a review. Aust Dent J.

[15] Beris A, Gkiatas I, Gelalis I, Papadopoulos D, Kostas-Agnantis I (2019). Current concepts in peripheral nerve surgery. Eur J Orthop Surg Traumatol.

[16] Gaudin R, Knipfer C, Henningsen A, Smeets R, Heiland M, Hadlock T (2016). Approaches to Peripheral Nerve Repair: Generations of Biomaterial Conduits Yielding to Replacing Autologous Nerve Grafts in Craniomaxillofacial Surgery. Biomed Res Int.

[17] Wolford LM, Rodrigues DB (2011). Autogenous grafts/allografts/conduits for bridging peripheral trigeminal nerve gaps. Atlas Oral Maxillofac Surg Clin North Am.

[18] Zuniga JR, LaBanc JP (1993). Advances in microsurgical nerve repair. Journal of Oral and Maxillofacial Surgery.

[19] Ziccardi VB, Assael LA (2001). Mechanisms of trigeminal nerve injuries. Atlas Oral Maxillofac Surg Clin North Am.

[20] Salomon D, Miloro M, Kolokythas A (2016). Outcomes of Immediate Allograft Reconstruction of Long-Span Defects of the Inferior Alveolar Nerve. Journal of Oral and Maxillofacial Surgery.

[21] Ray WZ, Mackinnon SE (2010). Management of nerve gaps: autografts, allografts, nerve transfers, and end-to-side neurorrhaphy. Exp Neurol.

[22] Bagheri SC, Meyer RA, Cho SH, Thoppay J, Khan HA, Steed MB (2012). Microsurgical repair of the inferior alveolar nerve: success rate and factors that adversely affect outcome. J Oral Maxillofac Surg.

[23] Miloro M, Stoner JA (2005). Subjective outcomes following sural nerve harvest. J Oral Maxillofac Surg.

[24] Staniforth P, Fisher TR (1978). The effects of sural nerve excision in autogenous nerve grafting. Hand.

[25] Rappaport WD, Valente J, Hunter GC, Rance NE, Lick S, Lewis T (1993). Clinical utilization and complications of sural nerve biopsy. Am J Surg.

[26] Pan D, Mackinnon SE, Wood MD (2020). Advances in the repair of segmental nerve injuries and trends in reconstruction. Muscle Nerve.

[27] Zuniga JR, Williams F, Petrisor D (2017). A Case-and-Control, Multisite, Positive Controlled, Prospective Study of the Safety and Effectiveness of Immediate Inferior Alveolar Nerve Processed Nerve Allograft Reconstruction With Ablation of the Mandible for Benign Pathology. J Oral Maxillofac Surg.

[28] Shanti RM, Ziccardi VB (2011). Use of decellularized nerve allograft for inferior alveolar nerve reconstruction: a case report. J Oral Maxillofac Surg.

[29] Safa B, Buncke G (2016). Autograft Substitutes: Conduits and Processed Nerve Allografts. Hand Clin.

[30] Le Donne M, Jouan R, Bourlet J, Louvrier A, Ducret M, Sigaux N (2022). Inferior alveolar nerve allogenic repair following mandibulectomy: A systematic review. J Stomatol Oral Maxillofac Surg.

[31] Ziccardi VB (2011). Microsurgical techniques for repair of the inferior alveolar and lingual nerves. Atlas Oral Maxillofac Surg Clin North Am.

